# Adverse events in clinically complex elderly patients with atrial fibrillation according to oral anticoagulation status

**DOI:** 10.1016/j.eclinm.2024.102974

**Published:** 2024-12-01

**Authors:** Tommaso Bucci, Giulio Francesco Romiti, Hironori Ishiguchi, Luigi Gerra, Marta Mantovani, Bi Huang, Marco Proietti, Gregory Y.H. Lip

**Affiliations:** a Liverpool Centre for Cardiovascular Science at University of Liverpool, Liverpool John Moores University and Liverpool and Heart and Chest Hospital, Liverpool, United Kingdom; b Department of Clinical Internal, Anaesthesiologic and Cardiovascular Sciences, Sapienza University of Rome, Rome, Italy; c Department of Translational and Precision Medicine, Sapienza, University of Rome, Rome, Italy; d Division of Cardiology, Department of Medicine and Clinical Science, Yamaguchi University Graduate School of Medicine, Japan; e Cardiology Division Department of Biomedical Metabolic and Neural Sciences, University of Modena and Reggio Emilia, Modena, Italy; f Department of Cardiology, The First Affiliated Hospital of Chongqing Medical University, Chongqing, China; g Department of Clinical Sciences and Community Health, University of Milan, Milan, Italy; h Division of Subacute Care, IRCCS Istituti Clinici Scientifici Maugeri, Milan, Italy; i Danish Center for Health Services Research, Department of Clinical Medicine, Aalborg University, Aalborg, Denmark

**Keywords:** Atrial fibrillation, Clinical complexity, Anticoagulants, Bleeding, Thromboembolism

## Abstract

**Background:**

Few data are available about the impact of oral anticoagulants (OAC) in patients with Atrial Fibrillation (AF) and clinical complexity (CC).

**Methods:**

We conducted a retrospective study utilising data from the TriNetX network. Based on ICD-10-CM codes entered between 2020 and 2022, AF patients aged ≥75 years on long-term OAC with CC were categorised into two groups based on OAC use in the year before entering the study (maintained vs discontinued). CC was defined as BMI ≤23 kg/m^2^, and/or history of bleeding, and/or chronic kidney disease. The primary outcomes were the one-year risk of all-cause death, major cardiovascular events (MACE), and major bleeding. Cox regression analyses were used to calculate hazard ratios (HRs) and 95% CIs before and after 1:1 propensity score matching (PSM).

**Findings:**

We identified 6554 AF CC patients who discontinued OAC (mean age 81.5 ± 6.0 years, 46.7% females) and 23,212 AF patients with CC who maintained OAC (81.3 ± 6.0 years, 49.4% females). Before PSM, AF CC patients who discontinued OAC had a higher prevalence of intracranial, gastrointestinal haemorrhages, and antiplatelet use, with no significant differences after PSM. OAC discontinuation was associated with a higher risk of all-cause death (HR 1.22, 95% CI 1.11–1.35) and MACE (HR 1.38, 95% CI 1.25–1.53). The one-year risk of major bleeding was similar in those who discontinued or maintained OAC (HR 1.05, 95% CI 0.94–1.18), although it was significantly higher during the early follow-up (HR 1.51, 95% CI 1.24–1.83). The risk of primary outcomes decreased over time, with the risk of bleeding becoming not significant.

**Interpretation:**

AF CC patients who discontinued OAC have a high risk of adverse events. New antithrombotic and integrated care approaches to reduce thrombotic risk without increasing bleeding risk are needed in these patients.

**Funding:**

This study received no funding.

**Research in context**

**Evidence before this study**

With the ageing of the general population, the overall prevalence of Atrial Fibrillation (AF) is increasing, as is the proportion of patients with AF and Clinical Complexity (CC). CC is defined as the coexistence of various Janus-faced factors, such as advanced age, low body weight, chronic kidney disease, and haemorrhagic diathesis, which can increase the risk of both thrombotic and haemorrhagic events. This dual risk causes uncertainties in the net clinical benefit of oral anticoagulants (OAC).

**Added value of this study**

OAC discontinuation in patients with AF and CC is associated with an increased risk of major adverse cardiovascular events and death. The risk of bleeding in patients who discontinued OAC is comparable to those who continued OAC, except during the early period, when the risk is higher, likely attributable to the reasons that influence the clinical decision of discontinuing OAC.

**Implications of all the available evidence**

New antithrombotic strategies and integrated care approaches to reduce thrombotic risk while mitigating bleeding risk are needed.

## Introduction

Atrial fibrillation (AF) is the most common arrhythmia worldwide and is associated with an increased risk of cardiovascular events and death.^1^ With the progressively ageing population, it is estimated that the overall prevalence of AF will increase, along with the proportion of AF patients with clinical complexity (CC), which includes factors such as advanced age, chronic kidney disease (CKD), low body mass index (BMI), and previous haemorrhagic events,^2^, ^3^, ^4^ all entailing a more impaired and frail health status. In AF patients, CC significantly increases the baseline risk of bleeding, which may potentially outweigh the net clinical benefit of oral anticoagulants (OAC) for thrombotic prevention.^5^ Additionally, AF patients with CC have a lower chance of being prescribed an OAC, and a higher risk of OAC discontinuation if already on antithrombotic treatments.^3^^,^^4^^,^^6^ However, very limited data are available on the trajectories and clinical course (including risk of mortality, thrombotic and haemorrhagic events) of AF patients with CC who discontinued OAC.

Hence, our aim was to assess the risk of adverse events in AF patients with CC based on OAC use or discontinuation.

## Methods

### Study design

This retrospective observational study was conducted using TriNetX. This global federated health research network provides access to electronic medical records from various participating healthcare organisations, including academic medical centres, specialty physician practices, and community hospitals. The network encompasses approximately 250 million individuals. The available data within this network include demographics, diagnoses using the International Classification of Diseases, Ninth and Tenth Revisions, Clinical Modification (ICD-9-CM and ICD-10-CM) codes, and medications coded with Veteran Affairs (VA) Codes. More information can be found online (https://trinetx.com/company-overview/).

TriNetX operates as a health research network in compliance with the Health Insurance Portability and Accountability Act (HIPAA) and U.S. federal law, ensuring the privacy and security of healthcare data, including de-identified data, as mandated by HIPAA's Privacy Rule. Access to TriNetX's data requires submitting requests to TriNetX and a data-sharing agreement. Studies conducted within the TriNetX research network do not require ethical approval or informed consent since they do not involve identifiable patient information, which aligns with its status as a federated research network. Additional details regarding data extraction from TriNetX are provided in the Supplementary Material.

### Cohort

The searches on the TriNetX online research platform were conducted on October 15, 2024, using the U.S. Collaborative Network. Based on recorded ICD-10-CM codes, we included individuals aged ≥75 years with AF on long-term anticoagulation who either continued or discontinued OAC (warfarin and non-vitamin K oral anticoagulants [NOAC]) use in the year preceding the start of the observation period (Supplementary Figure S1). The index event, marking the beginning of the observation period, was the first recording of the ICD code for AF (I48) between January 1, 2020, and December 31, 2022. At the time of the search, data from 64 participating healthcare organisations, exclusively located in the U.S., were available for patients who met the study inclusion criteria.

CC was defined as the coexistence of a low BMI ≤23 kg/m^2^ and/or CKD (stages III, IV, V, and end-stage renal disease), and/or previous bleeding episodes (I60, I61, I62: intracranial haemorrhages; K92.2, K92.1: gastrointestinal bleeding; and R58: haemorrhage, not elsewhere classified) occurring between the second and the third years prior to the entry into the observation period (Supplementary Figure S1). To avoid including patients with a first AF diagnosis between 2020 and 2022, a previous code for AF must have been recorded between two and three years prior to the index event.

### OAC use and discontinuation

Long-term OAC was defined as the presence of two instances of OAC: one between the second and the third years and the other between the first and second years prior to the entry into the observation period (Supplementary Figure S1). Based on the presence or absence of any codes related to OAC during the last year before the start of the observation period, we subdivided these patients into i) AF patients with CC who discontinued OAC, and ii) AF patients with CC who maintained OAC. Any other diagnoses or treatments reported within three years before the index event were considered the individual's baseline characteristics.

To avoid competition between the adverse events that occurred during the last year before the entry and those recorded during the observation period, and to standardize the exposure (OAC continuation and discontinuation) to one year, we excluded all patients who experienced the outcome of interest during the last year before of the study enter (Supplementary Figure S1).

More information about the ICD-10-CM codes utilised for the inclusion and exclusion criteria are found in Supplementary Table S1.

### Outcomes

The primary outcomes were the one-year risk of all-cause death, major adverse cardiovascular events (MACE: ischemic stroke, transient ischemic attack, peripheral arterial embolism, and myocardial infarction), and major bleeding (central nervous system [CNS], gastrointestinal [GI], internal bleeding, and hypovolemic shock). Secondary outcomes included each component of the primary outcome and catheter ablation. To quantify the significance of unmeasured bias and confounding related to “frail” status—beyond the differing prevalence of comorbidities—we considered an additional endpoint expected to be affected in frail patients, defined as “slipping, tripping, stumbling, and falls”.^7^ The adverse events of interest were identified via ICD-10-CM codes (Supplementary Table S2).

### Statistical analysis

Baseline characteristics of patients with CC who discontinued OAC and those who maintained OAC were balanced through logistic regression and propensity score matching (PSM) in a 1:1 ratio. The greedy nearest neighbour method was utilized, employing a calliper of 0.1 pooled standard deviations without replacement. The balance of demographic and clinical variables between the groups was assessed using Absolute Standardized Mean Differences (ASD), with an ASD of less than 0.1 indicating well-balanced characteristics. We included the following variables in the PSM: age, sex, ethnicity, hypertension, ischemic heart diseases, ischemic stroke, heart failure, diabetes, dyslipidaemia, obesity, peripheral artery disease, aortic aneurysm and dissection, atherosclerosis, chronic rheumatic heart diseases, pulmonary heart diseases, each feature of CC (CKD, low BMI, and previous haemorrhages), aplastic and other anaemias and other bone marrow syndromes, nutritional anaemias, coagulation defects, purpura and other haemorrhagic conditions, malignancies, liver cirrhosis, and cardiovascular medications (including β-blockers, antiarrhythmics, diuretics, statins, antianginals, calcium channel blockers, angiotensin-converting enzyme inhibitors, angiotensin II inhibitors, and antiplatelets). These variables were selected based on their potential association with the risk of thrombotic and haemorrhagic events. Cox proportional hazard models, before and after PSM, were used to calculate hazard ratios (HRs) and 95% confidence intervals (95% CI) for the risk of adverse events in AF patients with CC not on OAC compared to those on OAC.

To determine whether the proportional hazards assumption for primary outcomes was satisfied in the Cox regression models after PSM, we conducted a Chi-square (χ^2^) test based on Schoenfeld residuals. Further details on the performance and interpretation of this test can be found under the “Supplementary Methods” section of the Supplementary Material. When the proportional hazards assumption was not met in the primary analysis, we performed additional analyses analysing the one-year follow-up period into two phases: an early phase (the first 30 days) and a late phase (from day 31 to the end of the year). We then reassessed the risk using Cox regression and re-tested the proportional hazards assumption for each phase.

Sensitivity analyses were performed to validate and contextualise the results obtained from the main analysis. The risk of adverse events in patients with AF and CC who discontinued OAC, compared to those who maintained OAC, was assessed based on sex, the components defining clinical complexity, and the type of anticoagulant (warfarin or NOACs). For the analysis based on OAC type, the group of patients who discontinued OAC included only those prescribed warfarin or NOACs during the second and third years before the observation period. In the group of patients who maintained OAC, only those prescribed warfarin or NOACs continuously during the second and third years and the year prior to the observation period were included. Patients who switched to anticoagulant type (warfarin or NOAC) within three years before the observation period were excluded from the analysis.

Aalen-Johansen curves were used to represent the daily cumulative incidence of primary outcomes, addressing potential concerns about competing risks among the different primary outcomes in AF patients with CC, with or without OAC. The cumulative incidence was calculated as the number of new cases divided by the number of individuals at risk daily.

All analyses were conducted using the TriNetX statistical platform, which employs both R and Python for data analysis. Survival analyses were performed using the R Survival library (v3.2–3), while propensity risk scores were calculated through logistic regression implemented with the scikit-learn package in Python version 3.7. TriNetX does not impute or estimate clinical values to fill patient record gaps. All tests were two-tailed, with statistical significance defined as p-values less than 0.05, indicating a Type I error rate of less than 5% if the null hypothesis is true.

### Role of funding

This study received no funding.

## Results

**Fig. 1:**
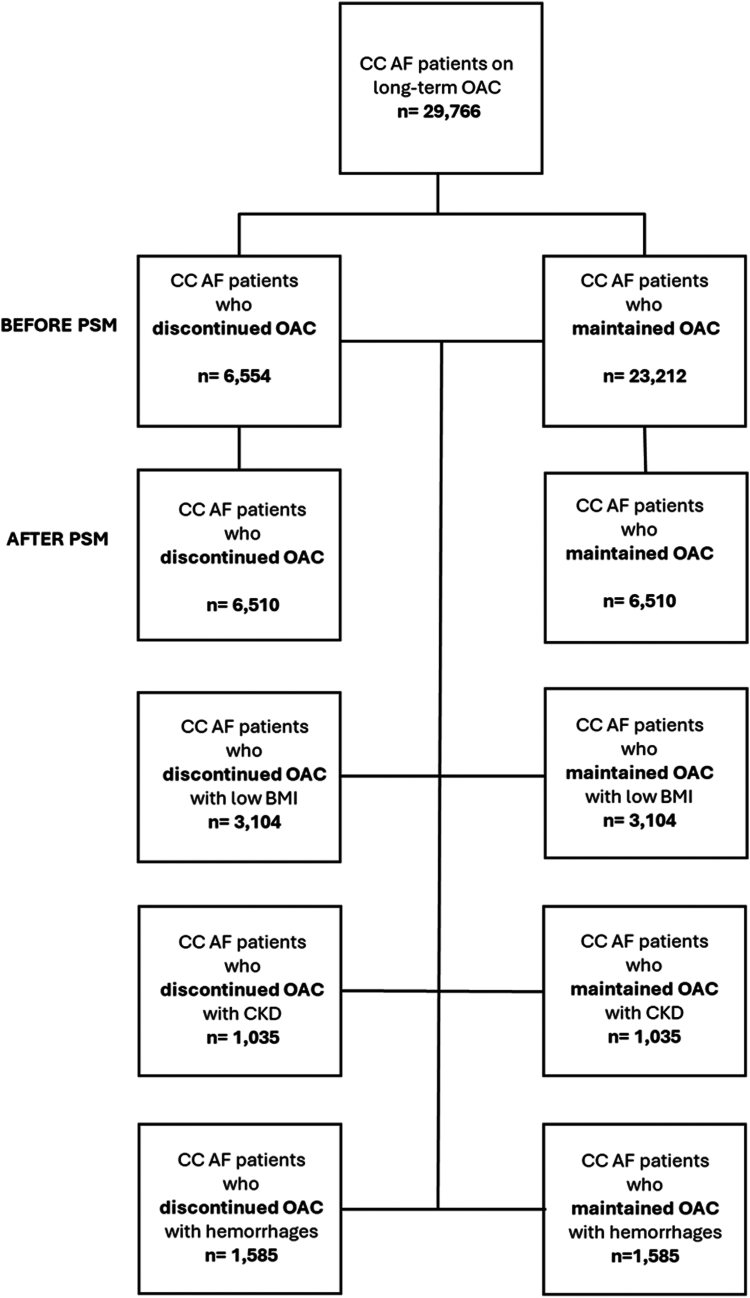
**Flow chart of the study**.

The final cohort consisted of 6554 AF patients with CC who discontinued OAC (mean age 81.5 ± 6.0, 46.7% females) and 23,212 patients with CC who continued OAC (mean age 81.3 ± 6.0 years, 49.4% females); study flow-chart is reported in Fig. 1. Before PSM, AF patients with CC who discontinued OAC had higher prevalences of intracranial haemorrhages, GI bleeding, and antiplatelets use but a lower prevalence of dyslipidaemia, pulmonary heart disease, and CKD stage III compared to AF patients with CC who maintained OAC (Table 1). During the year prior to the study entry, before PSM, among those who maintained OAC, 9402 (40.5%) were treated with warfarin, 11,935 (51.4%) with NOACs; 1875 (8.1%) did not have specification on the type of OAC received.

**Table 1:** Baseline characteristics of patients with atrial fibrillation and clinical complexity who discontinued oral anticoagulants compared to those who continued oral anticoagulants.

	Pre PSM			After PSM		
	AF CC patients who discontinued OAC (n = 6554)	AF CC patients who maintained OAC (n = 23,212)	ASD	AF CC patients who discontinued OAC (n = 6510)	AF CC patients who maintained OAC (n = 6510)	ASD
Age, mean ± SD	81.5 ± 6.0	81.3 ± 6.0	0.024	81.5 ± 6.0	81.5 ± 6.0	0.005
Female sex, n (%)	3063 (46.7)	11,473 (49.4)	0.058	3046 (46.8)	3068 (47.1)	0.007
White, n (%)	5162 (78.8)	18,743 (80.7)	0.049	5133 (78.8)	5125 (78.7)	0.003
Black or African, n (%)	375 (5.7)	1383 (6.0)	0.010	371 (5.7)	371 (5.7)	<0.001
Asian, n (%)	202 (3.1)	633 (2.7)	0.021	201 (3.1)	194 (3.0)	0.006
SBP, mean ± SD	129.4 ± 20.8	129.0 ± 19.6	0.018	129.4 ± 20.8	129.0 ± 19.7	0.027
DBP, mean ± SD	81.5 ± 6.0	81.5 ± 6.0	0.005	70.0 ± 12.0	70.3 ± 11.6	0.016
eGFR, mean ± SD	53.4 ± 22.3	52.3 ± 21.6	0.048	53.3 ± 22.3	52.9 ± 21.6	0.027
BMI, mean ± SD	26.8 ± 6.7	27.2 ± 6.9	0.072	26.8 ± 6.7	27.2 ± 6.9	0.007
BMI ≤23, n (%)	2994 (45.7)	10,273 (44.3)	0.029	2983 (45.8)	2951 (45.3)	0.010
Hypertension, n (%)	5768 (88.0)	20,595 (88.7)	0.022	5730 (88.0)	5729 (88.0)	<0.001
Diabetes, n (%)	2492 (38.0)	9019 (38.9)	0.017	2473 (38.0)	2459 (37.8)	0.004
Obesity, n (%)	1490 (22.7)	6054 (26.1)	0.078	1482 (22.8)	1447 (22.2)	0.013
Dyslipidaemia, n (%)	4832 (73.7)	18,217 (78.5)	0.112	4805 (73.8)	4845 (74.4)	0.014
CKD III stage, n (%)	3045 (46.5)	12,791 (55.1)	0.174	3040 (46.7)	3075 (47.2)	0.011
CKD IV stage, n (%)	714 (10.9)	3078 (13.3)	0.073	713 (11.0)	692 (10.6)	0.010
CKD V stage, n (%)	141 (2.2)	511 (2.2)	0.003	140 (2.2)	132 (2.0)	0.009
End stage renal disease, n (%)	141 (2.2)	511 (2.2)	0.040	333 (5.1)	318 (4.9)	0.011
Heart failure, n (%)	3529 (53.8)	13,139 (56.6)	0.056	3511 (53.9)	3563 (54.7)	0.016
Ischemic heart disease, n (%)	3682 (56.2)	13,084 (56.4)	0.004	3657 (56.2)	3694 (56.7)	0.011
Pulmonary heart disease, n (%)	1431 (21.8)	6334 (27.3)	0.127	1425 (21.9)	1450 (22.3)	0.009
Aortic aneurysm, n (%)	468 (7.1)	1870 (8.1)	0.035	465 (7.1)	475 (7.3)	0.006
Ischaemic stroke, n (%)	603 (9.2)	1599 (6.9)	0.085	583 (9.0)	596 (9.2)	0.007
Nontraumatic intracerebral haemorrhages, n (%)	153 (2.3)	177 (0.8)	0.128	125 (1.9)	130 (2.0)	0.006
Nontraumatic subarachnoid haemorrhages, n (%)	82 (1.3)	132 (0.6)	0.072	72 (1.1)	76 (1.2)	0.006
Other and unspecified nontraumatic intracranial haemorrhages, n (%)	198 (3.0)	337 (1.5)	0.106	174 (2.7)	172 (2.6)	0.002
Coagulation defects, n (%)	1184 (18.1)	4640 (20.0)	0.049	1174 (18.0)	1137 (17.5)	0.015
Aplastic and other anaemias, n (%)	3060 (46.7)	10,449 (45.0)	0.034	3032 (46.6)	2996 (46.0)	0.011
Nutritional anaemias, n (%)	1559 (23.8)	5586 (24.1)	0.007	1546 (23.7)	1495 (23.0)	0.019
Cirrhosis of liver, n (%)	158 (2.4)	523 (2.3)	0.010	158 (2.4)	156 (2.4)	0.002
Gastrointestinal haemorrhages, unspecified, n (%)	893 (13.6)	1943 (8.4)	0.169	880 (13.5)	892 (13.7)	0.005
Melena, n (%)	726 (11.1)	2057 (8.9)	0.074	722 (11.1)	743 (11.4)	0.010
Haemorrhage, not elsewhere classified, n (%)	225 (3.4)	925 (4.0)	0.029	225 (3.5)	221 (3.4)	0.005
Beta Blockers, n (%)	5129 (78.3)	18,689 (80.5)	0.056	5098 (78.3)	5069 (77.9)	0.011
Diuretics, n (%)	4276 (65.2)	16,004 (68.9)	0.079	4252 (65.3)	4289 (65.9)	0.012
Calcium Channel Blockers, n (%)	3209 (49.0)	11,483 (49.5)	0.010	3185 (48.9)	3187 (49.0)	0.001
Antiarrhythmics, n (%)	4419 (67.4)	15,130 (65.2)	0.047	4381 (67.3)	4367 (67.1)	0.005
ACE Inhibitors, n (%)	2022 (30.9)	8002 (34.5)	0.077	2010 (30.9)	1988 (30.5)	0.007
Sartans, n (%)	1761 (26.9)	6784 (29.2)	0.052	1752 (26.9)	1808 (27.8)	0.019
Antiplatelets, n (%)	3482 (52.3)	10,529 (45.4)	0.139	3393 (52.1)	3419 (52.5)	0.008

AF, Atrial Fibrillation; ASD, Absolute Standardized mean Difference; CC, Clinical Complexity; DBP, Diastolic Blood Pressure; NOAC, Non-vitamin K Antagonist Oral Anticoagulants; OAC, Oral Anticoagulants; SBP, Systolic Blood Pressure; SD, Standard Deviation.

The complete list of variables utilized for the propensity score matching can be found in Supplementary Table S3.

The number of primary and secondary outcomes and the relative HRs for the comparison before and after PSM are reported in Table 2. Before PSM, AF patients with CC who discontinued OAC demonstrated a higher risk of all-cause death (HR 1.23, 95% CI 1.14–1.32), MACE (HR 1.48, 95% CI 1.37–1.60) and major bleeding (HR 1.15, 95% CI 1.06–1.26) compared to those who maintained OAC. This higher risk in AF patients with CC who discontinued OAC was also observed in most of the secondary outcomes, with an increased risk of thromboembolism, myocardial infarction, CNS bleeding, and GI bleeding. No significant associations were found for the one-year risk of internal bleeding and hypovolemic shock (Table 2). Moreover, AF patients with CC who discontinued OAC had a lower likelihood of undergoing catheter ablation procedures than AF patients with CC who maintained OAC (HR 0.66, 95% CI 0.48–0.91) and a significantly increased risk of falls (HR 1.18, 95% CI 1.10–1.27) compared to those who maintained OAC.

**Table 2:** Risks of primary and secondary outcomes in patients with atrial fibrillation and clinical complexity who continued anticoagulant compared to those who discontinued anticoagulant.

	Before PSM			After PSM		
	Discontinued OAC n = 6554	Maintained OAC n = 23,212	HR (95% CI)	Discontinued OAC n = 6510	Maintained OAC n = 6510	HR (95% CI)
**All-cause death, n (%)**	884 (13.9)	2512 (12.0)	1.23 (1.14–1.32)	915 (14.1)	788 (12.1)	1.22 (1.11–1.35)
**MACE, n (%)**	895 (14.1)	2127 (10.1)	1.48 (1.37–1.60)	903 (13.9)	690 (10.6)	1.38 (1.25–1.53)
Thromboembolism, n (%)	521 (8.2)	1234 (5.9)	1.47 (1.33–1.63)	525 (8.1)	429 (6.6)	1.28 (1.13–1.45)
AMI, n (%)	431 (6.8)	1033 (4.9)	1.45 (1.29–1.62)	435 (6.7)	314 (4.8)	1.45 (1.25–1.68)
**Major Bleeding, n (%)**	641 (9.1)	2069 (8.3)	1.15 (1.06–1.26)	575 (8.8)	570 (8.8)	1.05 (0.94–1.18)
CNS bleeding, n (%)	114 (1.8)	294 (1.4)	1.34 (1.08–1.66)	114 (1.8)	102 (1.6)	1.16 (0.90–1.52)
GI bleeding, n (%)	411 (6.5)	1222 (5.8)	1.17 (1.04–1.30)	414 (6.4)	399 (6.1)	1.08 (0.94–1.24)
Internal bleeding, n (%)	16 (0.3)	53 (0.3)	1.05 (0.60–1.83)	16 (0.2)	18 (0.3)	0.93 (0.47–1.82)
Hypovolemic shock, n (%)	19 (0.3)	97 (0.5)	0.67 (0.41–1.10)	20 (0.3)	35 (0.5)	0.60 (0.34–1.03)
**Ablation procedures, n (%)**	44 (0.7)	231 (1.1)	0.66 (0.48–0.91)	47 (0.7)	89 (1.4)	0.55 (0.39–0.79)
**Falls, n (%)**	915 (14.4)	2706 (12.9)	1.18 (1.10–1.27)	906 (13.9)	778 (12.0)	1.23 (1.11–1.35)

AF, Atrial Fibrillation; CC, Clinical Complexity; CI, Confidence Interval; CNS, Central Nervous System; GI, Gastro-Intestinal; HR, Hazard Ratio; MACE, Major Cardiovascular Events; OAC, Oral Anticoagulants.

**Fig. 2:**
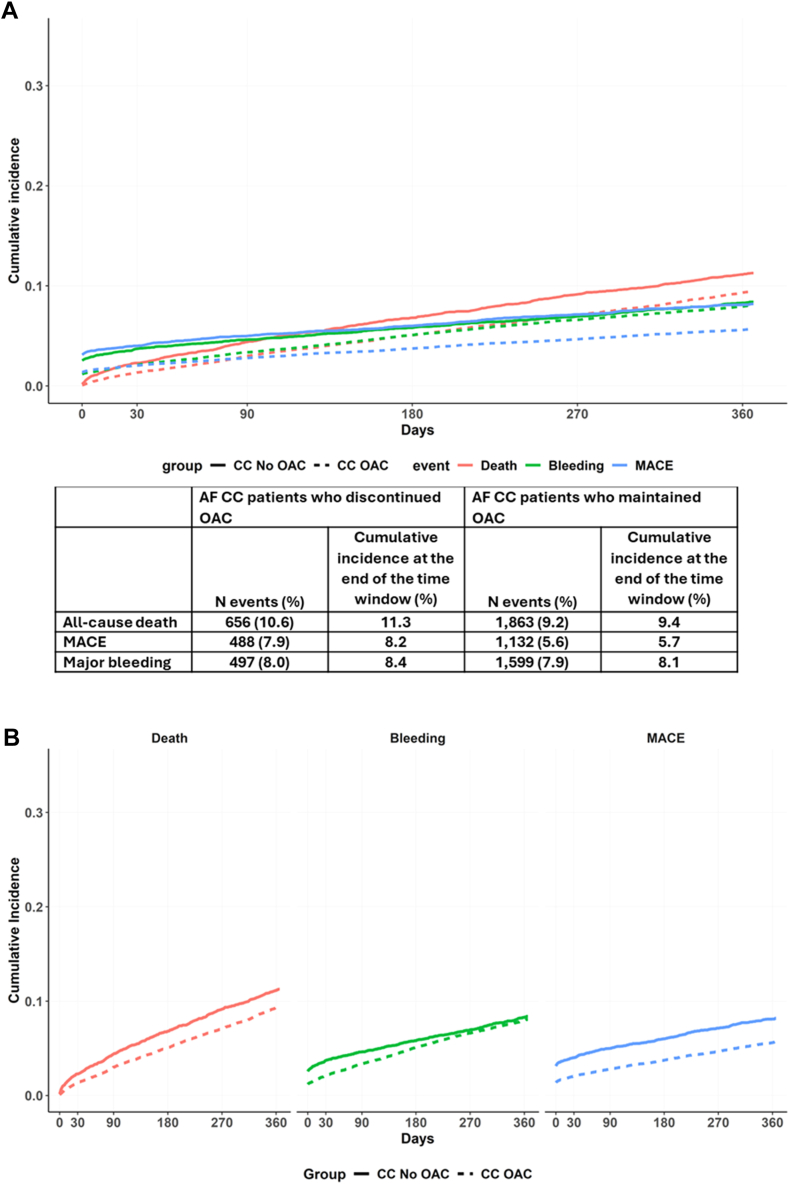
**Aalen-Johansen curves for the cumulative incidence of primary outcomes in patients with atrial fibrillation and clinical complexity based on oral anticoagulant use at baseline**. AF, Atrial Fibrillation; CC, Clinical Complexity; MACE, Major Adverse Cardiovascular Events; OAC, Oral Anticoagulants. The solid lines represent patients who discontinued oral anticoagulants, while the dashed lines represent patients who maintained oral anticoagulants. The colors in the graph are associated with different outcomes: red represents all-cause death, green represents major bleeding, and blue represents major adverse cardiovascular events.

Aalen–Johansen curves before PSM in AF patients with CC discontinuing and maintaining OAC are reported in Fig. 2. The one-year cumulative incidences for our primary outcomes were as follows: 11.3% and 9.4% for all-cause death; 8.2% and 5.7% for MACE; and 8.4% and 8.1% for major bleeding, respectively (Fig. 2, Panel A and B).

After PSM, we observed well balanced baseline characteristics between AF patients with CC who discontinued or continued OAC (Table 1, Supplementary Table S3). Compared to AF patients with CC maintaining OAC, those discontinuing OAC showed a significantly increased risk of all-cause death (HR 1.22, 95% CI 1.11–1.35) and MACE (1.38, 95% CI 1.25–1.53); no statistically significant difference was found for the risk of major bleeding (HR 1.05, 95% CI 0.94–1.18). Conversely, the higher risk of thromboembolism, myocardial infarction, as well as the lower probability of undergoing ablations procedures, and the increased risk of falls in AF patients with CC who discontinued OAC was confirmed even after PSM (Table 2). No statistically significant associations were found for the risk of CNS bleeding, and GI bleeding, internal bleeding, and hypovolemic shock (Table 2).

**Fig. 3:**
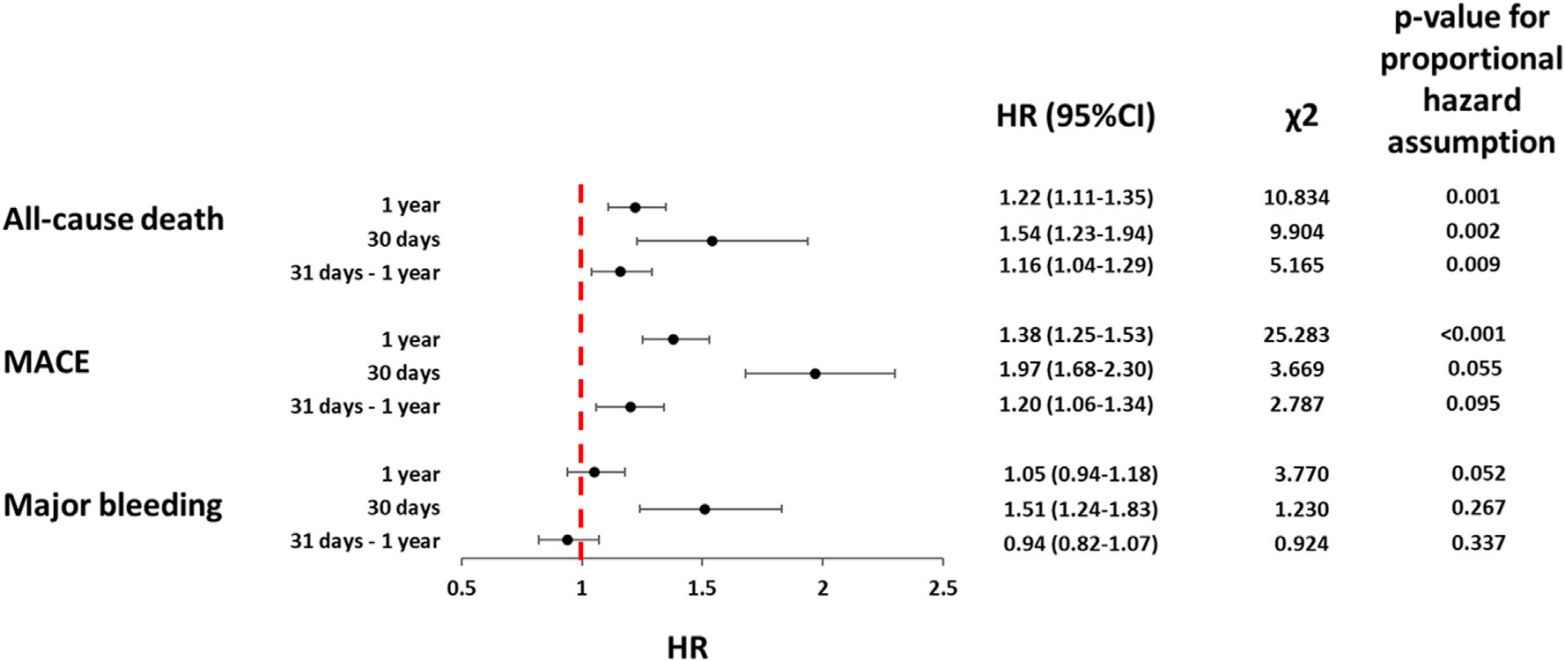
**Risk of primary outcomes in patients with atrial fibrillation and clinical complexity who discontinued oral anticoagulants in different time windows**. CI, Confidence Intervals; HR, Hazard Ratio; MACE, Major Adverse Cardiovascular Events. A high χ^2^ suggests a greater deviation from the expected values, indicating a potential violation of the proportional hazard assumption. Conversely, a small χ^2^ value indicates that the observed residuals closely match the expected values.

When we analysed the proportional hazard assumption for the one-year risk of primary outcomes in patients who discontinued OAC compared to those who maintained OAC after PSM, we found potential non-proportional hazards for all-cause death (χ^2^ 10.834), and MACE (χ^2^ 25.283), with borderline non-significant values for major bleeding (χ^2^ 3.770) (Fig. 3). When the follow-up period was subdivided, we observed that the risk of primary outcomes was of greater magnitude during the early vs late phase of follow-up: all-cause death (HR 1.54, 95% CI 1.23–1.94 vs HR 1.16, 95% CI 1.04–1.29 for early vs late period, respectively), MACE (HR 1.97, 95% CI 1.68–2.30 vs HR 1.20, 95% CI 1.06–1.34, for early vs late period, respectively), and major bleeding (HR 1.51, 95% CI 1.24–1.83 vs HR 0.94, 95% CI 0.82–1.07, for early vs late period, respectively) (Fig. 3).

The proportional hazard assumption during the early phase was satisfied for major bleeding (χ^2^ = 1.230) and MACE (χ^2^ = 3.669) but not for all-cause death (χ^2^ = 9.904). During the late follow-up phase, OAC discontinuation remained associated with a higher risk of all-cause death and MACE, whereas no statistically significant association was observed for major bleeding (Fig. 3). The proportional hazard assumption during the late phase was satisfied for major bleeding (χ^2^ = 1.230) and MACE (χ^2^ = 2.787), but not for all-cause death despite lower variability compared to the overall period (χ^2^ = 5.165).

### Sensitivity analyses

In the sensitivity analyses, we found that the higher risk of primary outcomes in AF patients with CC who discontinued OAC compared to those who maintained OAC was similar across sex and all prevalent CC manifestations (Table 3).

**Table 3:** Risk of primary outcomes in clinically relevant subgroups of patients.

Groups (n = number of patients after PSM)	All-cause death		MACE		Major Bleeding	
	N events	HR (95% CI)	N events	HR (95% CI)	N events	HR (95% CI)
**Sex**						
Discontinued OAC males (n = 2813)	425 (15.1)	1.23 (1.07–1.41)	401 (14.3)	1.38 (1.19–1.60)	276 (9.8)	1.10 (0.93–1.30)
Maintained OAC males (n = 2813)	367 (13.0)		310 (11.0)		264 (9.4)	
Discontinued OAC females (n = 2877)	364 (12.7)	1.37 (1.17–1.60)	393 (1.7)	1.37 (1.18–1.59)	237 (8.2)	1.09 (0.91–1.31)
Maintained OAC females (n = 2877)	285 (9.9)		305 (10.6)		229 (8.0)	
**Low BMI**						
Discontinued OAC with low BMI (n = 3104)	422 (15.1)	1.23 (0.99–1.54)	145 (16.6)	1.41 (1.01–1.80)	106 (12.2)	1.20 (0.91–1.58)
Continued OAC with low BMI (n = 3104)	309 (11.1)		111 (12.7)		95 (10.9)	
Discontinued OAC without low BMI (n = 3052)	454 (13.1)	1.17 (0.98–1.27)	510 (14.8)	1.44 (1.26–1.65)	359 (10.4)	1.10 (0.95–1.27)
Continued OAC without low BMI (n = 3052)	427 (12.4)		375 (10.9)		341 (9.9)	
**CKD**						
Discontinued OAC with CKD (n = 1035)	471 (15.2)	1.22 (1.07–1.39)	486 (15.7)	1.43 (1.25–1.63)	307 (9.9)	1.07 (0.91–1.26)
Continued OAC with CKD (n = 1035)	410 (13.2)		364 (11.7)		301 (9.7)	
Discontinued OAC without CKD (n = 3052)	388 (12.7)	1.29 (1.11–1.50)	392 (12.8)	1.28 (1.11–1.49)	255 (8.4)	1.03 (0.87–1.22)
Continued OAC without CKD (n = 3052)	320 (10.5)		323 (10.6)		261 (8.6)	
**Previous bleeding**						
Discontinued OAC with previous bleeding (n = 1585)	231 (14.6)	1.31 (1.08–1.59)	246 (15.5)	1.34 (1.11–1.62)	234 (15.0)	1.01 (0.84–1.20)
Continued OAC with previous bleeding (n = 1585)	186 (11.7)		194 (12.2)		248 (15.6)	
Discontinued OAC without previous bleeding (n = 4632)	640 (13.8)	1.26 (1.12–1.41)	639 (13.8)	1.45 (1.23–1.69)	326 (7.0)	1.15 (0.98–1.35)
Continued OAC without previous bleeding (n = 4632)	542 (11.7)		455 (9.8)		299 (6.5)	
**Type of OAC**						
Discontinued OAC with previous warfarin (n = 1034)	143 (13.8)	1.08 (0.85–1.36)	137 (13.2)	1.49 (1.15–1.94)	101 (9.8)	1.20 (0.90–1.59)
Continued OAC with warfarin (n = 1034)	138 (13.3)		97 (9.4)		88 (8.5)	
Discontinued OAC with NOACs (n = 2477)	327 (13.2)	1.48 (1.25–1.75)	352 (14.2)	1.66 (1.40–1.96)	192 (7.8)	1.09 (0.89–1.33)
Continued OAC with NOACs (n = 2477)	237 (9.6)		227 (9.2)		186 (7.5)	

BMI, Body Mass Index; CC, Clinical Complexity; CI, Confidence Interval; CKD, Chronic Kidney Disease; HR, Hazard Ratio; MACE, Major Cardiovascular Events; OAC, Oral Anticoagulant; PSM, Propensity Score Matching.

When analysing the risk of primary outcomes based on the type of OAC, we found that patients who discontinued warfarin, compared to those who continued it, had an increased risk of MACE, but no statistically significant differences were observed for all-cause death or major bleeding. Patients who discontinued NOACs had an increased risk of all-cause death and MACE compared to those who maintained them, with no significant difference in major bleeding (Table 3).

## Discussion

In this study, our main findings are as follows: 1) AF patients with CC who discontinued long-term OAC exhibited an increased risk of all-cause death and MACE along, without statistically significant differences in the risk of major bleeding; 2) AF patients with CC who discontinued OAC were also less likely undergoing ablation procedures compared to those who maintained OAC, and at higher risk of falling; 3) the risk of primary outcomes was highest in the initial phase of follow-up, when the risk of major bleeding was also significantly increased; however, the risk differences tended to be mitigated in the subsequent phase of follow-up, with risk of major bleeding becoming non-significant, while the risk of all-cause death and MACE, remained significantly elevated; 4) the increased risk of adverse events was similar across sex and prevalent CC manifestations; 4) both warfarin and NOACs discontinuations were associated with an increased risk of MACE and similar bleeding risk compared to patients who continued these treatments. Of note, NOACs discontinuation had a larger magnitude impact on the risk of all-cause death than warfarin discontinuation.

The observed higher risk of adverse events in AF patients with CC who discontinued OAC compared to those who maintained OAC underscores the complexity of selecting an appropriate antithrombotic regimen in this clinical scenario. In these patients, the various features of CC can present a dual challenge, contributing to both higher thromboembolic and bleeding risks, potentially leading to increased mortality through diverse mechanisms. For instance, the frail phenotype, characterised by low BMI and advanced age, may affect the metabolism of OACs and their distribution in plasma, predisposing patients to unforeseeable effects.^8^ CKD-related vascular calcification and metabolic abnormalities can promote both thrombosis and vascular rupture due to increased vessel stiffness,^9^ while a haemorrhagic diathesis may result in recurrent bleeding, albeit paradoxically activating the clotting system,^10^ potentially leading to fatal outcomes.

Furthermore, this is compounded by the frequent coexistence of CC features within individual patients, forming a distinct clinical phenotypic cluster that complicates the assessment of each CC component's specific contribution. Our sensitivity analysis, which revealed a consistent increase in the risk of adverse events when considering each CC component separately, supports this hypothesis. Additionally, in patients with AF and CC, it is crucial to consider not only the presence of comorbidities but also their differential impact on individuals. Among patients with the same risk factors, those discontinuing OAC treatment exhibited an increased risk of falls, suggesting a greater degree of frailty compared to those continuing OAC. This underscores the complexity of factors in assessing the net benefit of antithrombotic therapy. The potential futility of OAC treatment is influenced not only by thrombotic and haemorrhagic risks, but also by variations in individual functional status.

In our population, only 70% of AF patients with CC were prescribed OAC, highlighting the underuse of stroke prevention strategies, particularly given that all patients were aged ≥75 years and at high thromboembolic risk (CHA_2_DS_2_-VASc score ≥2). This likely accounts for the higher risk of MACE and all-cause death observed in patients who discontinued OAC, though the lack of difference in bleeding risk remains less clear. One possible explanation may be the persistence of the clinical reasons that initially led to the interruption of OAC treatment, suggesting that in this clinical scenario, the OAC underuse may reflect a clinical decision made after careful consideration of the net clinical balance between thromboembolic risk prevention, and the risk of potentially life-threatening bleeding. This hypothesis is supported by the high baseline cumulative incidence of major bleeding from the first day of observation and the higher risk estimates observed during the early follow-up phase compared to the late phase. Notably, the observation period began one year after OAC discontinuation, and we can hypothesise that the ICD code for AF was likely recorded in most cases during hospital admissions for acute events.

In our sensitivity analysis, comparing the risk of adverse events based on the type of OAC, we found a significantly increased risk of all-cause death and MACE, in those who discontinued NOACs compared to those who maintain this treatment. In contrast, while there was an increased risk of MACE, no significant association was found for the risk all-cause death in those who discontinued warfarin. This may suggest the presence of indication bias. Although the introduction of NOACs has improved the clinical management of AF patients,^11^ they are contraindicated in certain high-risk conditions, such as advanced liver cirrhosis, where warfarin is sometimes recommended. As a result, the baseline high risk of death in patients prescribed warfarin may have obscured the impact of OAC discontinuation on mortality, with death potentially being attributed to other causes rather than MACE. Conversely, in patients prescribed NOACs, OAC discontinuation may have a greater impact on the risk of all primary outcomes. However, this study was not designed to explore these factors, and further prospective research studies will be needed to clarify these issues.

The data from our study contrasts with a previous study that investigated the impact of OAC discontinuation in a cohort of 1578 AF patients aged ≥75 years from Italy.^12^ In this study, although OAC discontinuation was associated with an increased one-year risk of all-cause death, it was not associated with an increased risk of thromboembolism or major bleeding after adjustment for confounders. This discrepancy could be related to the relatively small sample size and the low overall incidence of thromboembolic (2.6%) and haemorrhagic events (4.7%) recorded during the study period, as well as the higher incidence of death observed in that cohort compared to ours, that may have caused a more substantial competing risk effect on non-fatal outcomes. As we have shown in our study, the risk of adverse events in this clinical context is dynamic and should be addressed in different time windows.

This clinical scenario is even more complicated when considering the implications on other management aspects. Indeed, the low likelihood of undergoing ablation procedures observed in AF patients with CC who discontinued OAC may contribute to the elevated risk of adverse events seen in these patients, given the superiority of early rhythm control over rate control in reducing the risk of adverse events and death in AF patients, even with multimorbidity.^13^^,^^14^ Several factors, including the perceived higher risk of adverse events such as serious bleeding,^15^ may have contraindicated these interventional procedures in some AF patients with CC at high bleeding risk and may explain these results. Indeed, the higher risk for falls found in AF patients with CC who discontinued OAC seem consistent with the hypothesis that the perceived baseline risk of these patients may have influenced both the interruption of OAC, the lower likelihood of interventional procedures, and the overall higher risk of adverse outcomes.

Our study underlines the need for new, tailored approaches to manage AF patients with CC, to reduce their thromboembolic risk while mitigating the risk of bleeding. For patients at high risk of thrombosis, where bleeding predisposition outweighs this risk, left atrial appendage (LAA) closure is another option.^16^, ^17^, ^18^ The 2024 European guidelines for AF management suggest using percutaneous LAA closure in patients with AF and a contraindication for long-term OAC to prevent thromboembolism, with a class IIb recommendation and level C evidence.^19^ However, the need for non-standardized antithrombotic regimens following the procedure continues to limit its widespread use in AF patients with concurrent conditions and a high risk of bleeding.^20^ Finally, the new factor XIa (FXIa) inhibitors, which are being evaluated in phase III trials, may offer a new approach for AF patients with CC^21^: epidemiological studies have shown that while increased levels of FXI are associated with a high risk of thrombotic events, its deficiency does not increase bleeding risk.^22^ Whilst encouraging results from phase II trials were evident,^23^ a recent phase III randomised clinical trial was prematurely interrupted due to the higher rates of thromboembolic events in patients treated with asundexian 50 mg daily compared to those receiving the standard dose of apixaban.^24^

All this evidence underscores the importance of an integrated and holistic approach in the clinical management of AF patients with CC, given their high risk of adverse events, and their complex health needs. Although the optimal antithrombotic approach in patients at high risk of bleeding is still debated, full adherence to the ABC (Atrial fibrillation Better Care) pathway is effective in improving outcomes for AF patients,^25^^,^^26^ even in those with CC^27^^,^^28^ or with low-educational status.^29^ In this regard, the AFFIRMO Programme, a multicentre ongoing study involving 20 different European health institutions, will provide novel evidence in applying the ABC pathway in conjunction with the comprehensive geriatric assessment.^30^ Indeed, this study aims to elucidate how specific care models can be implemented in the general older multimorbid AF population to improve clinical management and reduce the risks of major adverse clinical outcomes in this growing group of AF patients with CC.

Several limitations should be considered in the interpretation of our results. First, the retrospective nature raises the possibility of unmeasured and selection bias. Second, excluding patients who experienced the outcome of interest in the year before the observation period may have resulted in the omission of those at the highest risk of adverse events, potentially limiting the generalizability of our results. Third, administrative data may fail to identify a significant proportion of AF patients with CC or accurately reflect the dynamic changes in OAC use/non-use over time. Moreover, it may be prone to potential misdiagnosis. Fourth, our analysis considered only the use of OAC, so we cannot assess the impact of parenteral anticoagulants on the clinical course of AF patients with CC. Fifth, the competing risk analysis suggested a potential underestimation of the risk of MACE and major bleeding in AF patients with CC who maintained or discontinued OAC. Sixth, although the PSM balanced the two populations based on the prevalence of thrombotic and haemorrhagic risk factors, it did not adjust for the severity of these diseases. Seventh, we did not evaluate the impact of polypharmacy or potential drug interactions that may have influenced the risk of adverse events in patients with AF and CC. Eighth, outcomes that occurred outside the network may not have been well captured, potentially influencing the risk assessment. Finally, the study is limited by the inability to stratify the analysis according to ethnicity, the presence of social disparities, and the limited use of LAA closure.

In conclusion, AF CC patients who discontinued OAC have a high risk of adverse events. New antithrombotic approaches to reduce thrombotic risk without increasing bleeding risk are needed in these CC patients.

## Contributors

TB: conceptualisation, formal analysis, interpretation of results, writing original draft; IH, LG, MM, BH critically revised the manuscript; GFR and MP: conceptualisation, interpretation of results and writing original draft; GYHL: supervision, validation, and writing original draft. All authors approved the final version of the manuscript. TB accessed and verified the data underlying this study.

## Data sharing statement

The data utilized for this study are available on the TriNetX online platform. Access to TriNetX's data requires submitting requests to TriNetX and a data-sharing agreement.

## Declaration of interests

GFR reports consultancy for Boehringer Ingelheim and an educational grant from Anthos, outside the submitted work. No fees are directly received personally; MP is Italian national leader of the AFFIRMO project on multimorbidity in atrial fibrillation, which has received funding from the European Union's Horizon 2020 research and innovation program under grant agreement No 899871; GYHL is a consultant and speaker for BMS/Pfizer, Boehringer Ingelheim, Daiichi-Sankyo, Anthos. No fees are received personally. GYHL is a National Institute for Health and Care Research (NIHR) Senior Investigator and co-principal investigator of the AFFIRMO project on multimorbidity in AF, which has received funding from the European Union's Horizon 2020 research and innovation program under grant agreement No 89987. All other authors report no disclosures.
