# Supplementary material for: Adverse events in clinically complex elderly patients with atrial fibrillation according to oral anticoagulation status
Source: eClinicalMedicine. 2024 Dec 1;78:102974. doi: 10.1016/j.eclinm.2024.102974 (PMC11648801; doi:10.1016/j.eclinm.2024.102974)
Supplement: Supplementary material [file mmc1.docx]

**Adverse events in Clinically Complex elderly patients with Atrial Fibrillation according to oral anticoagulation status.**

Tommaso Bucci, Giulio Francesco Romiti, Ishiguchi Hironori, Luigi Gerra, Marta Mantovani, Bi Huang, Marco Proietti, Gregory Y.H. Lip

Supplementary material

***Supplementary Methods***

TriNetX Database

The TriNetX data are collected from member healthcare organizations (HCO) and originates from their primary electronic health records (EHR) system. A typical HCO is a large academic health center with data coming from majority of its affiliates. A single HCO frequently has more than one facility, including main and satellite hospitals. The data are stored on the TriNetX database via a physical server at the institution’s data centre or a virtual hosted appliance. The TriNetX platform comprises of a series of these appliances connected into a federated network. This network can broadcast queries to each appliance. Results are subsequently collected and aggregated. Once the data are sent to the network, it is mapped to a standard and controlled set of clinical terminologies and undergoes a data quality assessment including ‘data cleaning’ that rejects records which do not meet the TriNetX quality standards. The TriNetX database performs internal and extensive data quality assessment with every refresh based on conformance, completeness, and plausibility (http://doi.org/10.13063/2327-9214.1244). HIPAA (Health Insurance Portability and Accountability Act) compliance of the clinical patient data is achieved using deidentification. Available data types within the network include demographics, diagnoses (represented by ICD-10-CM codes), procedures (coded in ICD-10-PCS or CPT), and measurements (coded to LOINC). While extensive information is provided about patients’ diagnoses and procedures, other variables (such as socioeconomic and lifetime factors are not comprehensively represented). The advantage of EHR data over insurance claim data is that both insured and uninsured patients are included. An advantage of EHR data over survey data is that the former represents the diagnostic rates in the population presenting to healthcare facilities. This provides an accurate account of the burden of specific diagnoses on healthcare systems. One primary limitation of relying on diagnoses is that they do not account for undiagnosed patients who might have a condition but have not yet received medical support. Another general limitation of EHR data is that a patient may be seen in different HCO for different components of their care. If one HCO is not part of the federated network, then part of their medical records may not be available. Using a network of healthcare organizations, rather than a single site, limits this possibility but does not fully remove it.

Propensity Score Matched Analyses were performed using logistic regression [Logistic Regression from the scikit-learn package in Python (version 3.7)]. TriNetX performed a 1:1 greedy nearest neighbor matching model with a caliper of 0.1 pooled standard deviations. To eliminate bias resulting from nearest neighbor algorithms, the rows were randomized. Any baseline characteristic with a standardized mean difference between cohorts lower than 0.1 was deemed well matched. (<https://www.tandfonline.com/doi/full/10.1080/00273171.2011.568786>).

Assessment of the Proportional Hazards Assumption

To evaluate whether the proportional hazards assumption was satisfied in the Cox regression models, we conducted a Chi-square (χ²) test based on Schoenfeld residuals. These tests examine whether the relationship between the associated variables and the hazard function remains stable over time. The null hypothesis posits that the effect of OAC discontinuation on the hazards of primary outcomes is constant throughout the study period. The χ² statistic measures the discrepancy between the observed and expected Schoenfeld residuals. A higher χ² value indicates a greater divergence from the expected values, suggesting a potential violation of the proportional hazards assumption. Conversely, a lower χ² value implies that the observed residuals closely align with the expected values, supporting the assumption. The p-value, derived from the χ² statistic, reflects the likelihood of observing these deviations under the null hypothesis. A p-value greater than 0.05 suggests that the deviations are likely due to random variation, indicating that the proportional hazards assumption holds. In contrast, a p-value less than 0.05 implies that the observed deviations are unlikely to be random, indicating a violation of the proportional hazards assumption.

**Supplementary Figure 1**. Timeline of criteria used to create the two patient populations.


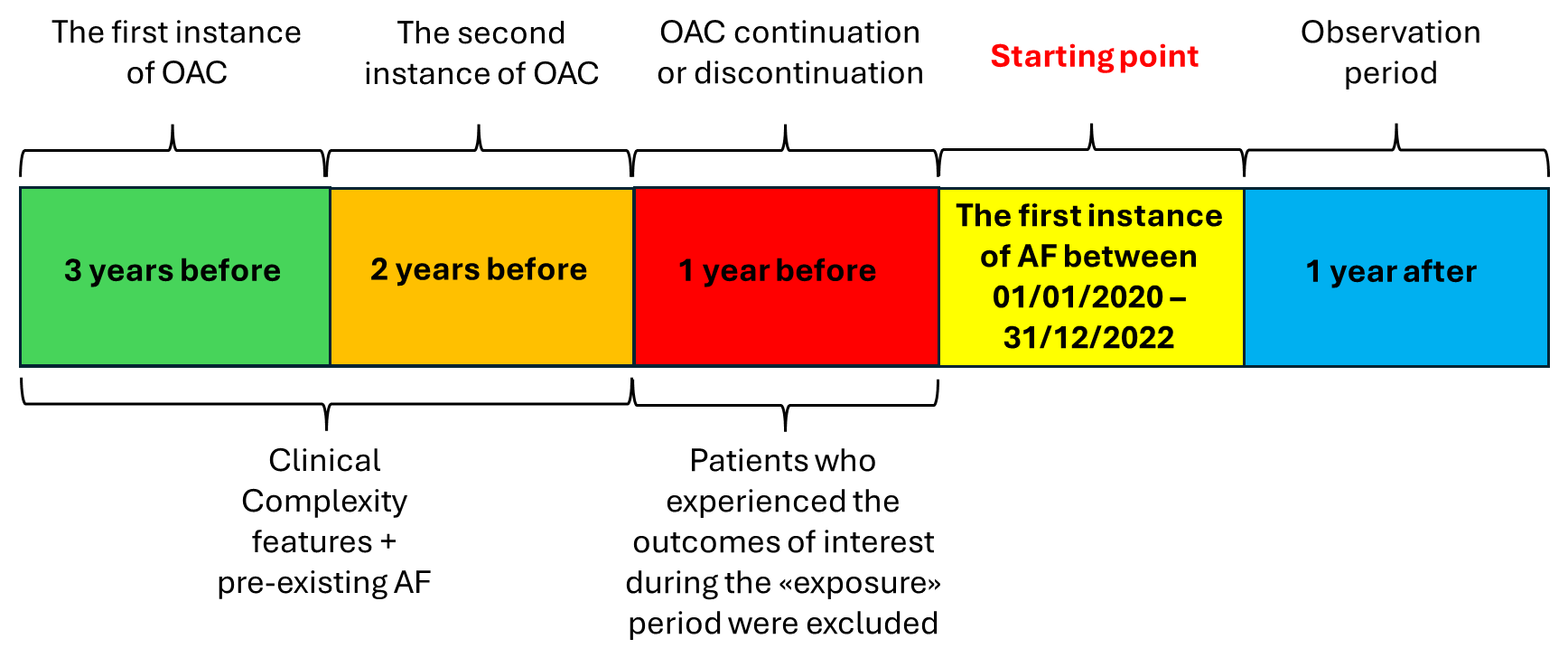


AF: Atrial Fibrillation, OAC: Oral Anticoagulants.

**Supplementary Table 1**. ICD-10-CM codes for inclusion and exclusion criteria in patients with atrial fibrillation with nonalcoholic fatty liver disease.

| ICD-10-CM, VA, LOINC, RxNorm and CPT codes | |
| --- | --- |
| **Patients with Atrial Fibrillation and Clinical Complexity who discontinued OAC** | |
| **Group 1** | |
| Inclusion criteria | First instance of Atrial Fibrillation (ICD code I48) reported in patients aged ≥ 75 years between 01st January 2020 – 31st December 2022 AND from 366 days to 1,095 days before, any instance of:   1. 1) Chronic Kidney Disease: stage III (ICD code N18.3), stage IV (ICD code N18.4), stage V (ICD code N18.5), and end stage renal disease (ICD code N18.6)   OR   1. 2) Body Mass Index ≤ 23 kg / m^2^ (LOINC 39156-5)   OR   1. 3) Previous bleeding: intracranial hemorrhage (ICD codes I60, I61, I61), Gastrointestinal bleeding (ICD codes K92.2 and K92.1), and unspecified hemorrhages (ICD code R58) 2. AND 3. 4) Atrial Fibrillation (ICD code I48) |
| Exclusion criteria | None |
| **Group 2** | |
| Inclusion criteria | First instance of Atrial Fibrillation (ICD code I48) reported in patients aged ≥ 75 years between 01st January 2020 – 31st December 2022 AND from 730 days (two years) to 1,095 (three years) days before, any instance of:  1) Warfarin (RxNorm 11289) OR edoxaban (RxNorm 1599538), OR rivaroxaban (RxNorm 1114195), OR dabigatran (RxNorm 1546356), OR apixaban (RxNorm 1364430) OR long term OAC (ICD code Z79.01) |
| Exclusion criteria | None |
| **Group 3** | |
| Inclusion criteria | First instance of Atrial Fibrillation (ICD code I48) reported in patients aged ≥ 75 years between 01st January 2020 – 31st December 2022 AND from 366 days to 729 days before, any instance of:  1) Warfarin (RxNorm 11289) OR edoxaban (RxNorm 1599538), OR rivaroxaban (RxNorm 1114195), OR dabigatran (RxNorm 1546356), OR apixaban (RxNorm 1364430) OR long term OAC (ICD code Z79.01) |
| Exclusion criteria | None |
| **Group 4** | |
| Inclusion criteria | None |
| Exclusion criteria | First instance of Atrial Fibrillation (ICD code I48) reported in patients aged ≥ 75 years between 01st January 2020 – 31st December 2022 AND from 1 days to 365 days before, any instance of:   1. Warfarin (RxNorm 11289) OR edoxaban (RxNorm 1599538), OR rivaroxaban (RxNorm 1114195), OR dabigatran (RxNorm 1546356), OR apixaban (RxNorm 1364430) OR long term OAC (ICD code Z79.01)   OR   1. Ischemic stroke (ICD code I63)   OR   1. Transient cerebral ischemic attacks (ICD code G45)   OR   1. Atheroembolism (ICD code I75)   OR   1. Central Nervous System hemorrhages (ICD codes I60, I61, I62, and S06.4)   OR   1. Internal bleeding (ICD codes J94.2 and K68.3)   OR   1. Hypovolemic shock (ICD code R57.1)   OR   1. Hemorrhage, not elsewhere classified (ICD code R58)   OR   1. Gastrointestinal bleeding (ICD codes K92.1, K92.2, K92.0) |
| **Patients with Atrial Fibrillation and Clinical Complexity who continued OAC** | |
| **Group 1** | |
| Inclusion criteria | First instance of Atrial Fibrillation (ICD code I48) reported in patients aged ≥ 75 years between 01st January 2020 – 31st December 2022 AND from 366 days to 1,095 days before, any instance of:   1. 1) Chronic Kidney Disease: stage III (N18.3), stage IV (N18.4), stage V (N18.5), and end stage renal disease (N18.6)   OR   1. 2) Body Mass Index ≤ 23 kg / m^2^ (LOINC 39156-5)   OR   1. 3) Previous bleeding: intracranial hemorrhage (ICD codes I60, I61, I61), Gastrointestinal bleeding (ICD codes K92.2 and K92.1), and unspecified hemorrhages (ICD code R58) 2. AND 3. 4) Atrial Fibrillation (ICD code I48) |
| Exclusion criteria | None |
| **Group 2** | |
| Inclusion criteria | First instance of Atrial Fibrillation (ICD code I48) reported in patients aged ≥ 75 years between 01st January 2020 – 31st December 2022 AND from 730 days (two years) to 1,095 (three years) days before, any instance of:  1) Warfarin (RxNorm 11289) OR edoxaban (RxNorm 1599538), OR rivaroxaban (RxNorm 1114195), OR dabigatran (RxNorm 1546356), OR apixaban (RxNorm 1364430) OR long term OAC (ICD code Z79.01) |
| Exclusion criteria | None |
| **Group 3** | |
| Inclusion criteria | First instance of Atrial Fibrillation (ICD code I48) reported in patients aged ≥ 75 years between 01st January 2020 – 31st December 2022 AND from 366 days to 729 days before, any instance of:  1) Warfarin (RxNorm 11289) OR edoxaban (RxNorm 1599538), OR rivaroxaban (RxNorm 1114195), OR dabigatran (RxNorm 1546356), OR apixaban (RxNorm 1364430) OR long term OAC (ICD code Z79.01) |
| Exclusion criteria | None |
| **Group 4** | |
| Inclusion criteria | First instance of Atrial Fibrillation (ICD code I48) reported in patients aged ≥ 75 years between 01st January 2020 – 31st December 2022 AND from 1 days to 365 days before, any instance of:   1. Warfarin (RxNorm 11289) OR edoxaban (RxNorm 1599538), OR rivaroxaban (RxNorm 1114195), OR dabigatran (RxNorm 1546356), OR apixaban (RxNorm 1364430) OR long term OAC (ICD code Z79.01) |
| Exclusion criteria | First instance of Atrial Fibrillation (I48) reported in patients aged ≥ 75 years between 01st January 2020 – 31st December 2022 AND from 1 days to 365 days before, any instance of:   1. Ischemic stroke (ICD code I63)   OR   1. Transient cerebral ischemic attacks (ICD code G45)   OR   1. Atheroembolism (ICD code I75)   OR   1. Central Nervous System hemorrhages (ICD codes I60, I61, I62, and S06.4)   OR   1. Internal bleeding (ICD codes J94.2 and K68.3)   OR   1. Hypovolemic shock (ICD code R57.1)   OR   1. Hemorrhage, not elsewhere classified (ICD code R58)   OR   1. Gastrointestinal bleeding (ICD codes K92.1, K92.2, K92.0) |

**Supplementary Table 2.** ICD-10-CM codes for the 1-year risk of all-cause death, thrombotic events, and bleeding.

|  | Diagnosis, ICD-10-CM, and CPT codes |
| --- | --- |
| **Primary outcomes** | |
| All-cause death | - Deceased (TriNetX variable) |
| Thromboembolism | - Ischemic stroke (ICD code I63)   OR   - Transient cerebral ischemic attacks (ICD code G45)   OR   - Atheroembolism (ICD code I75) |
| Major bleeding | - Central Nervous System hemorrhages (ICD codes I60, I61, I62, and S06.4)   OR   - Internal bleeding (ICD code J94.2 and K68.3)   OR   - Hypovolemic shock (ICD code R57.1)   OR   - Hemorrhage, not elsewhere classified (ICD code R58)   OR   - Gastrointestinal bleeding (ICD codes K92.1, K92.2, K92.0) |
| **Secondary outcomes** | |
| All-cause death | - Deceased (TriNetX variable) |
| Major Adverse Cardiovascular Events | - Ischemic stroke (ICD code I63)   OR   - Transient cerebral ischemic attacks (ICD code G45)   OR   - Atheroembolism (ICD code I75)   OR   - Acute Myocardial Infarction (ICD code I21) |
| Ablation procedures | - Comprehensive electrophysiologic evaluation with insertion and repositioning of multiple electrode catheters, induction or attempted induction of an arrhythmia with right atrial pacing and recording, and catheter ablation of arrhythmogenic focus, including intracardiac electrophysiologic 3-dimensional mapping, right ventricular pacing and recording, left atrial pacing and recording from coronary sinus or left atrium, and His bundle recording, when performed treatment of supraventricular tachycardia by ablation of fast or slow atrioventricular pathway, accessory atrioventricular connection, cavo-tricuspid isthmus or other single atrial focus or source of atrial re-entry (CPT 93653).   OR   - Comprehensive electrophysiologic evaluation including insertion and repositioning of multiple electrode catheters with induction or attempted induction of an arrhythmia with right atrial pacing and recording, right ventricular pacing and recording (when necessary), and His bundle recording (when necessary) with intracardiac catheter ablation of arrhythmogenic focus; with treatment of supraventricular tachycardia by ablation of fast or slow atrioventricular pathway, accessory atrioventricular connection, cavo-tricuspid isthmus or other single atrial focus or source of atrial re-entry (CPT 93654).   OR   - Comprehensive electrophysiologic evaluation including transseptal catheterizations, insertion and repositioning of multiple electrode catheters with intracardiac catheter ablation of atrial fibrillation by pulmonary vein isolation, including intracardiac electrophysiologic 3-dimensional mapping, intracardiac echocardiography including imaging supervision and interpretation, induction or attempted induction of an arrhythmia including left or right atrial pacing/recording, right ventricular pacing/recording, and His bundle recording, when performed (CPT 93656). |
| Falls | - Slipping, tripping, stumbling and falls (ICD code W00-W19) |

**Supplementary Table 3.** Baseline characteristics of patients with atrial fibrillation and clinical complexity who discontinued oral anticoagulants compared to those who continued oral anticoagulants, both before and after propensity score matching.

| **Cohort 1 and cohort 2 patient count before and after propensity score matching** | | | | | | | | | | | | |
| --- | --- | --- | --- | --- | --- | --- | --- | --- | --- | --- | --- | --- |
|  | | | Cohort | | | Patient count before matching | | | | Patient count after matching | | |
|  | | | 1 – AF CC who discontinued OAC | | | 6,554 | | | | 6,510 | | |
|  | | | 2 – AF CC who maintained OAC | | | 23,212 | | | | 6,510 | | |
| **Propensity score density function - Before and after matching (cohort 1 - purple, cohort 2 - green)** | | | | | | | | | | | | |
|  |  | | 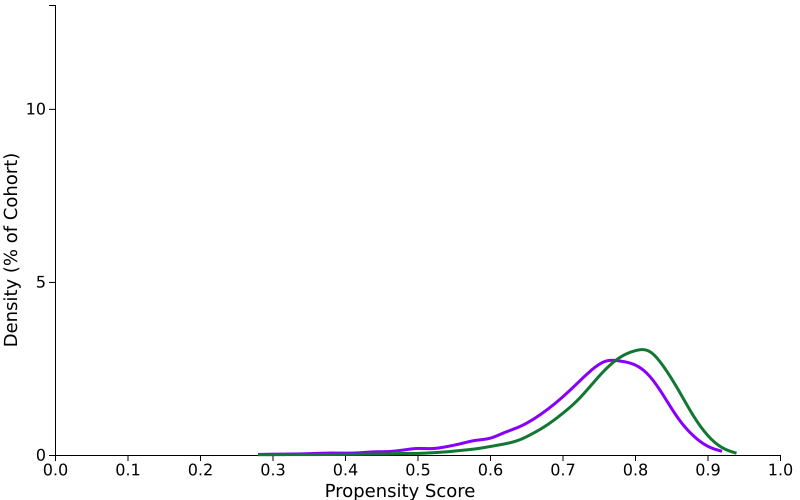 | | | | 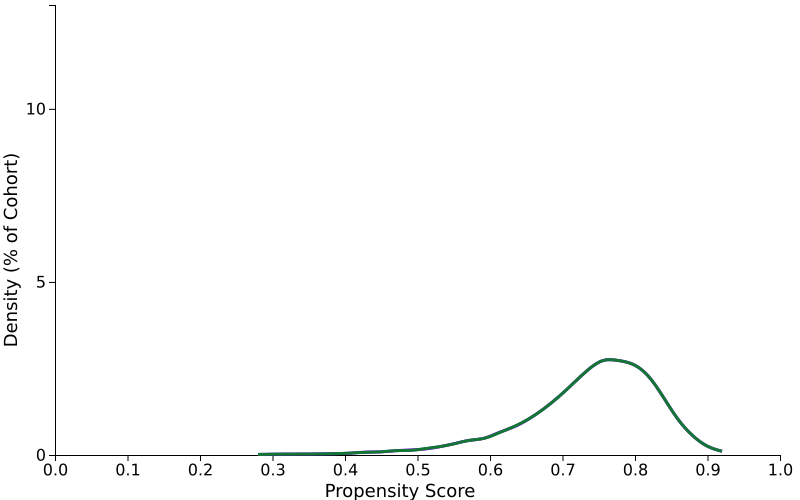 | | | | | |
| **Cohort 1 (N = 6,554) and cohort 2 (N = 23,212) characteristics before propensity score matching** | | | | | | | | | | | | |
|  | **Demographics** | | | | | | | | | | | |
|  |  | Cohort | | |  | Mean ± SD | | Patients | % of Cohort | | P-Value | Std diff. |
|  |  | 1 2 | | AI | Age at Index | 81.5 +/- 6.0 81.3 +/- 6.0 | | 6,554 23,212 | 100% 100% | | 0.087 | 0.024 |
|  |  | 1 2 | | 2106-3 | White |  | | 5,162 18,743 | 78.8% 80.7% | | <0.001 | 0.049 |
|  |  | 1 2 | | F | Female |  | | 3,063 11,473 | 46.7% 49.4% | | <0.001 | 0.054 |
|  |  | 1 2 | | 2054-5 | Black or African American |  | | 375 1,383 | 5.7% 6.0% | | 0.473 | 0.010 |
|  |  | 1 2 | | 2028-9 | Asian |  | | 202 633 | 3.1% 2.7% | | 0.124 | 0.021 |
|  | **Diagnosis** | | | | | | | | | | | |
|  |  | Cohort | | |  | Mean ± SD | | Patients | % of Cohort | | P-Value | Std diff. |
|  |  | 1 2 | | I10-I1A | Hypertensive diseases |  | | 5,768 20,595 | 88.0% 88.7% | | 0.107 | 0.022 |
|  |  | 1 2 | | I20-I25 | Ischemic heart diseases |  | | 3,682 13,084 | 56.2% 56.4% | | 0.786 | 0.004 |
|  |  | 1 2 | | E08-E13 | Diabetes mellitus |  | | 2,492 9,019 | 38.0% 38.9% | | 0.222 | 0.017 |
|  |  | 1 2 | | E66 | Overweight and obesity |  | | 1,490 6,054 | 22.7% 26.1% | | <0.001 | 0.078 |
|  |  | 1 2 | | E78 | Disorders of lipoprotein metabolism and other lipidemias |  | | 4,832 18,217 | 73.7% 78.5% | | <0.001 | 0.112 |
|  |  | 1 2 | | I50 | Heart failure |  | | 3,529 13,139 | 53.8% 56.6% | | <0.001 | 0.056 |
|  |  | 1 2 | | D60-D64 | Aplastic and other anemias and other bone marrow failure syndromes |  | | 3,060 10,449 | 46.7% 45.0% | | 0.016 | 0.034 |
|  |  | 1 2 | | D50-D53 | Nutritional anemias |  | | 1,559 5,586 | 23.8% 24.1% | | 0.642 | 0.007 |
|  |  | 1 2 | | D65-D69 | Coagulation defects, purpura and other hemorrhagic conditions |  | | 1,184 4,640 | 18.1% 20.0% | | 0.001 | 0.049 |
|  |  | 1 2 | | K74 | Fibrosis and cirrhosis of liver |  | | 158 523 | 2.4% 2.3% | | 0.451 | 0.010 |
|  |  | 1 2 | | C43-C44 | Melanoma and other malignant neoplasms of skin |  | | 472 2,474 | 7.2% 10.7% | | <0.001 | 0.121 |
|  |  | 1 2 | | C76-C80 | Malignant neoplasms of ill-defined, other secondary and unspecified sites |  | | 228 836 | 3.5% 3.6% | | 0.636 | 0.007 |
|  |  | 1 2 | | C60-C63 | Malignant neoplasms of male genital organs |  | | 317 1,353 | 4.8% 5.8% | | 0.002 | 0.044 |
|  |  | 1 2 | | C30-C39 | Malignant neoplasms of respiratory and intrathoracic organs |  | | 132 489 | 2.0% 2.1% | | 0.643 | 0.007 |
|  |  | 1 2 | | C64-C68 | Malignant neoplasms of urinary tract |  | | 189 793 | 2.9% 3.4% | | 0.033 | 0.030 |
|  |  | 1 2 | | C81-C96 | Malignant neoplasms of lymphoid, hematopoietic and related tissue |  | | 213 880 | 3.2% 3.8% | | 0.040 | 0.029 |
|  |  | 1 2 | | C15-C26 | Malignant neoplasms of digestive organs |  | | 190 706 | 2.9% 3.0% | | 0.551 | 0.008 |
|  |  | 1 2 | | C50-C50 | Malignant neoplasms of breast (C50) |  | | 209 962 | 3.2% 4.1% | | <0.001 | 0.051 |
|  |  | 1 2 | | C51-C58 | Malignant neoplasms of female genital organs |  | | 44 211 | 0.7% 0.9% | | 0.065 | 0.027 |
|  |  | 1 2 | | C00-C14 | Malignant neoplasms of lip, oral cavity and pharynx |  | | 43 120 | 0.7% 0.5% | | 0.178 | 0.018 |
|  |  | 1 2 | | C45-C49 | Malignant neoplasms of mesothelial and soft tissue |  | | 32 120 | 0.5% 0.5% | | 0.773 | 0.004 |
|  |  | 1 2 | | C73-C75 | Malignant neoplasms of thyroid and other endocrine glands |  | | 36 132 | 0.5% 0.6% | | 0.853 | 0.003 |
|  |  | 1 2 | | I63 | Cerebral infarction |  | | 603 1,599 | 9.2% 6.9% | | <0.001 | 0.085 |
|  |  | 1 2 | | I73 | Other peripheral vascular diseases |  | | 1,082 4,223 | 16.5% 18.2% | | 0.002 | 0.044 |
|  |  | 1 2 | | I70 | Atherosclerosis |  | | 1,083 4,236 | 16.5% 18.2% | | 0.001 | 0.046 |
|  |  | 1 2 | | I71 | Aortic aneurysm and dissection |  | | 468 1,870 | 7.1% 8.1% | | 0.015 | 0.035 |
|  |  | 1 2 | | I62 | Other and unspecified nontraumatic intracranial hemorrhage |  | | 198 337 | 3.0% 1.5% | | <0.001 | 0.106 |
|  |  | 1 2 | | I61 | Nontraumatic intracerebral hemorrhage |  | | 153 177 | 2.3% 0.8% | | <0.001 | 0.128 |
|  |  | 1 2 | | I60 | Nontraumatic subarachnoid hemorrhage |  | | 82 132 | 1.3% 0.6% | | <0.001 | 0.072 |
|  |  | 1 2 | | I26-I28 | Pulmonary heart disease and diseases of pulmonary circulation |  | | 1,431 6,334 | 21.8% 27.3% | | <0.001 | 0.127 |
|  |  | 1 2 | | I05-I09 | Chronic rheumatic heart diseases |  | | 1,401 5,923 | 21.4% 25.5% | | <0.001 | 0.098 |
|  |  | 1 2 | | K92.2 | Gastrointestinal hemorrhage, unspecified |  | | 893 1,943 | 13.6% 8.4% | | <0.001 | 0.169 |
|  |  | 1 2 | | K92.1 | Melena |  | | 726 2,057 | 11.1% 8.9% | | <0.001 | 0.074 |
|  |  | 1 2 | | R58 | Hemorrhage, not elsewhere classified |  | | 225 925 | 3.4% 4.0% | | 0.041 | 0.029 |
|  |  | 1 2 | | N18.3 | Chronic kidney disease, stage 3 (moderate) |  | | 3,045 12,791 | 46.5% 55.1% | | <0.001 | 0.174 |
|  |  | 1 2 | | N18.4 | Chronic kidney disease, stage 4 (severe) |  | | 714 3,078 | 10.9% 13.3% | | <0.001 | 0.073 |
|  |  | 1 2 | | N18.6 | End stage renal disease |  | | 334 988 | 5.1% 4.3% | | 0.004 | 0.040 |
|  |  | 1 2 | | N18.5 | Chronic kidney disease, stage 5 |  | | 141 511 | 2.2% 2.2% | | 0.807 | 0.003 |
|  | **Medication** | | | | | | | | | | | |
|  |  | Cohort | | |  | Mean ± SD | | Patients | % of Cohort | | P-Value | Std diff. |
|  |  | 1 2 | | CV100 | BETA BLOCKERS/RELATED |  | | 5,129 18,689 | 78.3% 80.5% | | <0.001 | 0.056 |
|  |  | 1 2 | | CV700 | DIURETICS |  | | 4,276 16,004 | 65.2% 68.9% | | <0.001 | 0.079 |
|  |  | 1 2 | | CV350 | ANTILIPEMIC AGENTS |  | | 4,206 15,628 | 64.2% 67.3% | | <0.001 | 0.066 |
|  |  | 1 2 | | CV200 | CALCIUM CHANNEL BLOCKERS |  | | 3,209 11,483 | 49.0% 49.5% | | 0.468 | 0.010 |
|  |  | 1 2 | | CV300 | ANTIARRHYTHMICS |  | | 4,419 15,130 | 67.4% 65.2% | | 0.001 | 0.047 |
|  |  | 1 2 | | CV800 | ACE INHIBITORS |  | | 2,022 8,002 | 30.9% 34.5% | | <0.001 | 0.077 |
|  |  | 1 2 | | CV805 | ANGIOTENSIN II INHIBITOR |  | | 1,761 6,784 | 26.9% 29.2% | | <0.001 | 0.052 |
|  |  | 1 2 | | BL117 | PLATELET AGGREGATION INHIBITORS |  | | 3,428 10,529 | 52.3% 45.4% | | <0.001 | 0.139 |
|  | **Laboratory** | | | | | | | | | | | |
|  |  | Cohort | | |  | Mean ± SD | | Patients | % of Cohort | | P-Value | Std diff. |
|  |  | 1 2 | | 9086 | Blood Pressure, Diastolic | 70.0 +/- 12.0 70.2 +/- 11.4 | | 4,684 16,308 | 71.5% 70.3% | | 0.203 | 0.021 |
|  |  | 1 2 | | 9083 | BMI | 26.8 +/- 6.7 27.2 +/- 6.9 | | 5,263 19,350 | 80.3% 83.4% | | <0.001 | 0.072 |
|  |  | 1 2 | |  | 0 - 23 kg/m2 |  | | 2,994 10,273 | 45.7% 44.3% | | 0.040 | 0.029 |
|  |  | 1 2 | | 9085 | Blood Pressure, Systolic | 129.4 +/- 20.8 129.0 +/- 19.6 | | 4,965 17,035 | 75.8% 73.4% | | 0.258 | 0.018 |
|  |  | 1 2 | | 8001 | Glomerular filtration rate/1.73 sq M.predicted [Volume Rate/Area] in Serum, Plasma or Blood by Creatinine-based formula (MDRD) | 53.4 +/- 22.3 52.3 +/- 21.6 | | 5,752 21,032 | 87.8% 90.6% | | 0.001 | 0.048 |
| **Cohort 1 (N = 6,510) and cohort 2 (N = 6,510) characteristics after propensity score matching** | | | | | | | | | | | | |
|  | **Demographics** | | | | | | | | | | | |
|  |  | Cohort | | |  | Mean ± SD | | Patients | % of Cohort | | P-Value | Std diff. |
|  |  | 1 2 | | AI | Age at Index | 81.5 +/- 6.0 81.5 +/- 6.0 | | 6,510 6,510 | 100% 100% | | 0.758 | 0.005 |
|  |  | 1 2 | | 2106-3 | White |  | | 5,133 5,125 | 78.8% 78.7% | | 0.864 | 0.003 |
|  |  | 1 2 | | F | Female |  | | 3,046 3,068 | 46.8% 47.1% | | 0.699 | 0.007 |
|  |  | 1 2 | | 2054-5 | Black or African American |  | | 371 371 | 5.7% 5.7% | | 1 | <0.001 |
|  |  | 1 2 | | 2028-9 | Asian |  | | 201 194 | 3.1% 3.0% | | 0.721 | 0.006 |
|  | **Diagnosis** | | | | | | | | | | | |
|  |  | Cohort | | |  | Mean ± SD | | Patients | % of Cohort | | P-Value | Std diff. |
|  |  | 1 2 | | I10-I1A | Hypertensive diseases |  | | 5,730 5,729 | 88.0% 88.0% | | 0.978 | <0.001 |
|  |  | 1 2 | | I20-I25 | Ischemic heart diseases |  | | 3,657 3,694 | 56.2% 56.7% | | 0.513 | 0.011 |
|  |  | 1 2 | | E08-E13 | Diabetes mellitus |  | | 2,473 2,459 | 38.0% 37.8% | | 0.800 | 0.004 |
|  |  | 1 2 | | E66 | Overweight and obesity |  | | 1,482 1,447 | 22.8% 22.2% | | 0.463 | 0.013 |
|  |  | 1 2 | | E78 | Disorders of lipoprotein metabolism and other lipidemias |  | | 4,805 4,845 | 73.8% 74.4% | | 0.424 | 0.014 |
|  |  | 1 2 | | I50 | Heart failure |  | | 3,511 3,563 | 53.9% 54.7% | | 0.360 | 0.016 |
|  |  | 1 2 | | D60-D64 | Aplastic and other anemias and other bone marrow failure syndromes |  | | 3,032 2,996 | 46.6% 46.0% | | 0.527 | 0.011 |
|  |  | 1 2 | | D50-D53 | Nutritional anemias |  | | 1,546 1,495 | 23.7% 23.0% | | 0.291 | 0.019 |
|  |  | 1 2 | | D65-D69 | Coagulation defects, purpura and other hemorrhagic conditions |  | | 1,174 1,137 | 18.0% 17.5% | | 0.396 | 0.015 |
|  |  | 1 2 | | K74 | Fibrosis and cirrhosis of liver |  | | 158 156 | 2.4% 2.4% | | 0.909 | 0.002 |
|  |  | 1 2 | | C43-C44 | Melanoma and other malignant neoplasms of skin |  | | 470 468 | 7.2% 7.2% | | 0.946 | 0.001 |
|  |  | 1 2 | | C76-C80 | Malignant neoplasms of ill-defined, other secondary and unspecified sites |  | | 224 222 | 3.4% 3.4% | | 0.923 | 0.002 |
|  |  | 1 2 | | C60-C63 | Malignant neoplasms of male genital organs |  | | 316 301 | 4.9% 4.6% | | 0.536 | 0.011 |
|  |  | 1 2 | | C30-C39 | Malignant neoplasms of respiratory and intrathoracic organs |  | | 130 123 | 2.0% 1.9% | | 0.657 | 0.008 |
|  |  | 1 2 | | C64-C68 | Malignant neoplasms of urinary tract |  | | 187 174 | 2.9% 2.7% | | 0.488 | 0.012 |
|  |  | 1 2 | | C81-C96 | Malignant neoplasms of lymphoid, hematopoietic and related tissue |  | | 209 206 | 3.2% 3.2% | | 0.881 | 0.003 |
|  |  | 1 2 | | C15-C26 | Malignant neoplasms of digestive organs |  | | 189 192 | 2.9% 2.9% | | 0.876 | 0.003 |
|  |  | 1 2 | | C50-C50 | Malignant neoplasms of breast (C50) |  | | 209 209 | 3.2% 3.2% | | 1 | <0.001 |
|  |  | 1 2 | | C51-C58 | Malignant neoplasms of female genital organs |  | | 44 49 | 0.7% 0.8% | | 0.603 | 0.009 |
|  |  | 1 2 | | C00-C14 | Malignant neoplasms of lip, oral cavity and pharynx |  | | 43 45 | 0.7% 0.7% | | 0.831 | 0.004 |
|  |  | 1 2 | | C45-C49 | Malignant neoplasms of mesothelial and soft tissue |  | | 32 30 | 0.5% 0.5% | | 0.799 | 0.004 |
|  |  | 1 2 | | C73-C75 | Malignant neoplasms of thyroid and other endocrine glands |  | | 35 40 | 0.5% 0.6% | | 0.563 | 0.010 |
|  |  | 1 2 | | I63 | Cerebral infarction |  | | 583 596 | 9.0% 9.2% | | 0.691 | 0.007 |
|  |  | 1 2 | | I73 | Other peripheral vascular diseases |  | | 1,075 1,068 | 16.5% 16.4% | | 0.869 | 0.003 |
|  |  | 1 2 | | I70 | Atherosclerosis |  | | 1,078 1,050 | 16.6% 16.1% | | 0.507 | 0.012 |
|  |  | 1 2 | | I71 | Aortic aneurysm and dissection |  | | 465 475 | 7.1% 7.3% | | 0.735 | 0.006 |
|  |  | 1 2 | | I62 | Other and unspecified nontraumatic intracranial hemorrhage |  | | 174 172 | 2.7% 2.6% | | 0.913 | 0.002 |
|  |  | 1 2 | | I61 | Nontraumatic intracerebral hemorrhage |  | | 125 130 | 1.9% 2.0% | | 0.752 | 0.006 |
|  |  | 1 2 | | I60 | Nontraumatic subarachnoid hemorrhage |  | | 72 76 | 1.1% 1.2% | | 0.741 | 0.006 |
|  |  | 1 2 | | I26-I28 | Pulmonary heart disease and diseases of pulmonary circulation |  | | 1,425 1,450 | 21.9% 22.3% | | 0.597 | 0.009 |
|  |  | 1 2 | | I05-I09 | Chronic rheumatic heart diseases |  | | 1,393 1,457 | 21.4% 22.4% | | 0.175 | 0.024 |
|  |  | 1 2 | | K92.2 | Gastrointestinal hemorrhage, unspecified |  | | 880 892 | 13.5% 13.7% | | 0.759 | 0.005 |
|  |  | 1 2 | | K92.1 | Melena |  | | 722 743 | 11.1% 11.4% | | 0.560 | 0.010 |
|  |  | 1 2 | | R58 | Hemorrhage, not elsewhere classified |  | | 225 221 | 3.5% 3.4% | | 0.847 | 0.003 |
|  |  | 1 2 | | N18.3 | Chronic kidney disease, stage 3 (moderate) |  | | 3,040 3,075 | 46.7% 47.2% | | 0.539 | 0.011 |
|  |  | 1 2 | | N18.4 | Chronic kidney disease, stage 4 (severe) |  | | 713 692 | 11.0% 10.6% | | 0.553 | 0.010 |
|  |  | 1 2 | | N18.6 | End stage renal disease |  | | 333 318 | 5.1% 4.9% | | 0.546 | 0.011 |
|  |  | 1 2 | | N18.5 | Chronic kidney disease, stage 5 |  | | 140 132 | 2.2% 2.0% | | 0.624 | 0.009 |
|  | **Medication** | | | | | | | | | | | |
|  |  | Cohort | | |  | Mean ± SD | | Patients | % of Cohort | | P-Value | Std diff. |
|  |  | 1 2 | | CV100 | BETA BLOCKERS/RELATED |  | | 5,098 5,069 | 78.3% 77.9% | | 0.539 | 0.011 |
|  |  | 1 2 | | CV700 | DIURETICS |  | | 4,252 4,289 | 65.3% 65.9% | | 0.495 | 0.012 |
|  |  | 1 2 | | CV350 | ANTILIPEMIC AGENTS |  | | 4,178 4,223 | 64.2% 64.9% | | 0.410 | 0.014 |
|  |  | 1 2 | | CV200 | CALCIUM CHANNEL BLOCKERS |  | | 3,185 3,187 | 48.9% 49.0% | | 0.972 | 0.001 |
|  |  | 1 2 | | CV300 | ANTIARRHYTHMICS |  | | 4,381 4,367 | 67.3% 67.1% | | 0.794 | 0.005 |
|  |  | 1 2 | | CV800 | ACE INHIBITORS |  | | 2,010 1,988 | 30.9% 30.5% | | 0.676 | 0.007 |
|  |  | 1 2 | | CV805 | ANGIOTENSIN II INHIBITOR |  | | 1,752 1,808 | 26.9% 27.8% | | 0.271 | 0.019 |
|  |  | 1 2 | | BL117 | PLATELET AGGREGATION INHIBITORS |  | | 3,393 3,419 | 52.1% 52.5% | | 0.648 | 0.008 |
|  | **Laboratory** | | | | | | | | | | | |
|  |  | Cohort | | |  | Mean ± SD | | Patients | % of Cohort | | P-Value | Std diff. |
|  |  | 1 2 | | 9086 | Blood Pressure, Diastolic | 70.0 +/- 12.0 70.3 +/- 11.6 | | 4,663 4,525 | 71.6% 69.5% | | 0.292 | 0.022 |
|  |  | 1 2 | | 9083 | BMI | 26.8 +/- 6.7 26.8 +/- 6.7 | | 5,232 5,427 | 80.4% 83.4% | | 0.708 | 0.007 |
|  |  | 1 2 | |  | 0 - 23 kg/m2 |  | | 2,983 2,951 | 45.8% 45.3% | | 0.573 | 0.010 |
|  |  | 1 2 | | 9085 | Blood Pressure, Systolic | 129.4 +/- 20.8 129.0 +/- 19.7 | | 4,943 4,773 | 75.9% 73.3% | | 0.433 | 0.016 |
|  |  | 1 2 | | 8001 | Glomerular filtration rate/1.73 sq M.predicted [Volume Rate/Area] in Serum, Plasma or Blood by Creatinine-based formula (MDRD) | 53.3 +/- 22.3 53.9 +/- 22.3 | | 5,715 5,834 | 87.8% 89.6% | | 0.151 | 0.027 |
